# Occupational prestige and future sickness absence and disability pension in women and men: a Swedish nationwide prospective cohort study

**DOI:** 10.1177/14034948241272936

**Published:** 2024-09-05

**Authors:** Gunnel Hensing, Mira Müller, Ylva Ulfsdotter Eriksson, Kristina Alexanderson, Kristin Farrants

**Affiliations:** 1 School of Public Health and Community Medicine, Institute of Medicine, Sahlgrenska Academy, University of Gothenburg, Sweden; 2 Division of Insurance Medicine, Department of Clinical Neuroscience, Karolinska Institutet, Stockholm, Sweden; 3 Department of Sociology and Work Science, University of Gothenburg, Sweden; 4 Department of Social Studies, Linnaeus University, Växjö, Sweden

**Keywords:** Occupational prestige, sick-leave, disability pension

## Abstract

**Background:**

Little is known about associations between occupational prestige, that is, the symbolic evaluation and social positioning of occupations, and sickness absence (SA) or disability pension (DP). We explored whether occupational prestige was associated with future SA or DP among women and men.

**Methods:**

A Swedish 4-year prospective cohort study of all those in paid work and aged 25–59 in 2010 (*N* = 2,605,227; 47% women), using linked microdata from three nationwide registers and Standard International Occupational Prestige Scale values, categorised as ‘very low’, ‘low’, ‘medium’, ‘high’, or ‘very high’. Odds ratios (ORs), 95% confidence intervals (CIs), crude and adjusted for several sociodemographic factors, were calculated for three outcomes: at least one SA spell (>14 days), >90 SA days, or DP occurrence, during follow-up (2011–2013).

**Results:**

The mean number of SA days in 2010 varied by occupational prestige group, for example, ‘very high’: 3.0, ‘very low’: 6.5. Compared to those in occupations with ‘very high’ prestige, all other groups had higher adjusted ORs for all three outcomes. Among men, those with ‘very low’ occupational prestige had the highest OR for at least one SA spell: OR 1.51 (95% CI 1.47–1.56); among women, the ‘medium’ group had the highest OR: 1.30 (1.27–1.32). The results were similar for SA >90 days. OR for DP among women with ‘very low’ occupational prestige was 2.01 (1.84–2.19), and 3.55 (3.15–4.01) for men.

**Conclusions:**

**Working in lower occupational prestige occupations was generally associated with higher odds of future SA/DP than working in higher prestige occupations; these associations were stronger for men than for women.**

## Introduction

There is a social gradient in both sickness absence (SA) and disability pension (DP): people in blue-collar occupations have higher levels [1]. The most prominent explanation is differences in the work environment, but this does not fully explain the social gradient [2]. Wilkinson and Pickett showed that being at the lower end of the social gradient may lead to feelings of misrecognition and stress, through internalisation of a negative social valuation of blue-collar jobs, low education, and a lower material standard [3]. This study explored to what extent the symbolic evaluation of occupations is associated with SA and DP.

Through collective value systems, occupations are ascribed different symbolic values, usually captured in the concept of ‘occupational prestige’. Occupational prestige scores (according to the Standard International Occupational Prestige Scale; SIOPS) are based on survey studies about people’s assessments of occupations’ social standing in society [4]. While socio-economic status (SES) taps into objective economic conditions, subjective social status (SSS) is the self-assessment of social position, in which occupational prestige taps into non-material symbolic evaluations of desirability and esteem [5,6]. Associations between SES, SSS and health and well-being are well documented. Less is known about associations between occupational prestige positioning and SA and DP.

So far, only one study, by Nwaru et al., has analysed occupational prestige scores in relation to future SA days [7]. Compared with the reference group (i.e. high occupational prestige; adjusted for survey year, gender, marital status, previous SA) the medium occupational prestige group had an incidence rate ratio (IRR) of 1.53 (95% CI 1.50–1.55), and the low occupational prestige group an IRR of 2.35 (95% CI 2.32–2.39). These IRRs attenuated after further adjustment for education and income but remained significant; however, when also adjusting for occupational class, employment type, contract type, and employment sector the results were non-significant. In an analysis of long-term SA (defined as >120 days), both women and men in lower prestige occupations had higher IRRs that persisted following adjustment. Those findings suggest that occupational prestige is associated with and contributes to the social gradient of SA.

However, the study by Nwaru et al. differed in certain ways to the current study. Nwaru et al. used survey data from a sample of employed people in Sweden, however, selection bias was possible [7]. Conversely, a nationwide register study including all those employed facilitates the drawing of more firm conclusions. Furthermore, occupational prestige was categorised into three groups [7], whereas using five groups, as in the current research, might give more detailed results. Finally, the previous study did not include information on DP as a possible outcome.

### Aim

The aim of the current study was to explore associations between occupational prestige and future SA and DP among women and men in paid work.

## Methods

A population-based prospective cohort study was conducted, with 2010 as the baseline and a 3-year follow-up.

### Data

We used pseudo-anonymised microdata from three nationwide registers, linked at the individual level by use of the personal identity number [8]. The Longitudinal Integration Database for Health Insurance and Labour Market Studies (LISA) held by Statistics Sweden [9] was used for identifying the cohort and for information on sex, age, educational level, income, occupation, type of living area, country of birth, and emigration. The Cause of Death Register held by the National Board of Health and Welfare was consulted for data on year of death [10]. Information on SA (i.e. SA spells >14 days) and DP (i.e. start- and end-dates and degree: full- or part-time) was obtained from the MicroData for Analysis of the Social Insurance database (MiDAS) [11] held by the Swedish Social Insurance Agency.

### The Swedish public sickness absence insurance system

In Sweden, people aged ⩾16 years, with income from work or unemployment benefits and whose work capacity is reduced due to morbidity, are eligible for SA benefits [12]. During the first 14 days of an SA spell, benefits are paid by the employer, then by the Social Insurance Agency. Thus, we did not include information on SA spells ⩽14 days. People aged 19–64 can be granted DP if their work capacity is long-term or permanently reduced due to morbidity. SA benefits cover about 80% and DP 65% of lost income, both up to a certain level. The standard age for old-age pension was 65 in 2010. Both SA and DP can be granted for full- or part-time (100%, 75%, 50% or 25% of ordinary work hours), and people can be on partial SA and DP at the same time. Therefore, we also calculated net days (e.g. two gross days of 50% absence = one net day).

### Study cohort

**Figure 1:**
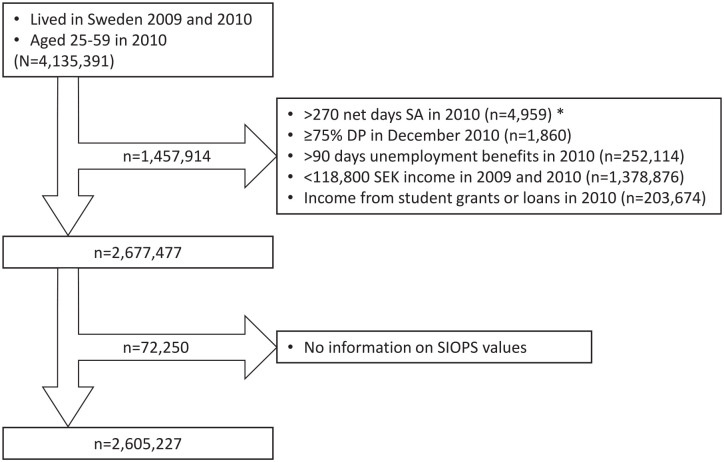
Flowchart of the study population. *Frequencies (*n*) were calculated separately for each exclusion criteria listed. There is a certain overlap between the frequencies within this box as it was possible for an individual to fulfil more than one exclusion criteria at the same time.

The study population included all individuals aged 25–59 in 2010, who had lived in Sweden throughout 2010, had no public student grants or loans, did not have >90 days of unemployment benefits, or >270 net SA days in 2010 and had an income from work ⩾118,800 SEK in both 2009 and 2010. We further excluded individuals who were on DP for ⩾75% in December 2010 to exclude individuals not at risk of SA. We included individuals who could be assigned a SIOPS value based on their occupational code registered in 2010, to ensure that they could be given an exposure value. This gave a cohort of 2,605,227 individuals (Figure 1).

We used the SIOPS to measure occupational prestige [4, 7, 13-16]. SIOPS is an international ranking classification where each occupation is given a score based on several rankings assigned by individuals in surveys in >60 countries [17]. SIOPS is based on the International Standard Classification of Occupations, revision 1988 (ISCO-88) [18]. We mapped categorisations of occupations according to the Swedish Standard Classifications of Occupations, revision 1996 (SSYK-96, the Swedish version of ISCO-88) into the corresponding occupations according to ISCO-88 and applied the SIOPS value to each individual according to their occupation. For more information on this mapping, see Nwaru et al. 2021 [7]. We categorised occupational prestige into five occupational prestige groups [19]: very low (SIOPS values 13–25, including occupations such as childminder, cleaner, porter, bartender, forester, fisher, and pawnbroker), low (26–38, e.g. receptionist, cashier, truck driver, machine operator, bricklayer, and assistant librarian), medium (39–51, e.g. nursery teacher, bank officer and credit counsellor, electrical engineer and electrical technician, motor vehicle repair staff, police, nurse assistant, nurse, and goldsmith), high (52–64, e.g. specialist nurse, librarian, pharmacist, teacher, data operator, social worker and counsellor, engineer officer, manager, and accountant), and very high (65–78, e.g. civil engineer, mathematician, lawyer, physician, pharmacologist, university lecturer, and architect).

We used the following measures of SA and DP [20, 21]: mean SA and DP net days/year, for all, for women and men, and for each of the occupational prestige groups, respectively. We also created three dichotomised variables that indicated whether a person, during the 3-year follow-up, had (1) any SA spell >14 gross days, (2) any SA spell >90 net days, and (3) any days with DP.

As covariates we used sex: woman, man; age: 25–34, 35–44, 45–54, 55–59 years; birth country: Sweden, other Nordic country, other EU27, rest of the world, or missing (for logistic regression analyses the categories ‘other Nordic country’ and ‘other EU27’ were combined into ‘Nordic and EU countries’, and missing was included in ‘rest of the world’; educational level: compulsory school (⩽9 years. In the analyses, ‘missing’ was included here), high school (10–12 years), or college/university (⩾13 years); family situation: married/cohabiting with children at home, married cohabiting without children at home, single with children at home, or single without children at home (‘children at home’ here means children <18 years of age living at home); type of living area: large city, medium-sized town, or small town/rural, based on the European classification degree of urbanisation (DEGURBA) [22]; and type of occupation: white-collar, blue-collar.

### Statistical analyses

We calculated descriptive statistics for the year of cohort inclusion and the mean annual number of SA and DP net days, respectively, in 2011–2013 for each occupational prestige group. Less than 1% of the cohort died or emigrated during follow-up (they were included in the analyses).

We used logistic regression to estimate crude and adjusted odds ratios (ORs) with 95% confidence intervals (CIs) for the two SA outcomes and the DP outcome. For adjusted ORs in Model I, all the covariates specified above were included, except ‘type of work’ (blue- or white collar), as we wanted to avoid over-adjustment. For adjusted ORs in Model II, all the covariates were included. All analyses were conducted for all and stratified by sex.

## Results

In the cohort of 2,605,227 people, 46.5% were women. The majority had at least some high school education (90.2%) and were born in Sweden (88.1%) (Table I). For an overview of the sociodemographic characteristics by occupational prestige group, see Supplementary Table 1.

**Table I.** Frequencies and proportions of sociodemographic characteristics in 2010, for all and stratified by sex.

Variables		All		Women		Men	
		*N*	%	*n*	%	*n*	%
	Total	2,605,227	100	1 210 144	100	1 395 083	100
Sex	Women	1,210,144	46.5	-	-	-	-
	Men	1,395,083	53.5	-	-	-	-
Level of education	Elementary (<10 years)	247,872	9.5	84 152	7.0	163 720	11.7
	High school (10–12 years)	1,266,289	48.6	550 233	45.5	716 056	51.3
	College/university (>12 years)	1,083,883	41.6	573 923	47.4	509 960	36.6
	Missing	7,183	0.3	1 836	0.2	5 347	0.4
Family situation	Married/cohabitant without children at home	536,017	20.60	293 343	24.2	242 674	17.4
	Married/cohabitant with children at home	1,064,896	40.9	458 409	37.9	606 487	43.5
	Single without children at home	864,828	33.2	354 168	29.3	510 660	36.6
	Single with children at home	139,486	5.4	104 224	8.6	35 262	2.5
Age (years)	25–34	543,077	20.8	218 365	18.0	324 712	23.3
	35–44	842,855	32.4	384 828	31.8	458 027	32.8
	45–54	842,630	32.3	417 094	34.5	425 536	30.5
	55–59	376,665	14.5	189 857	15.7	186 808	13.4
Birth country	Sweden	2,294,350	88.1	1 058 358	87.5	1 235 992	88.6
	Nordic country (except Sweden)	64,511	2.5	35 833	3.0	28 678	2.1
	EU27 (except Denmark, Finland, and Sweden)	59,446	2.3	28 362	2.3	31 084	2.2
	Rest of the world	186,869	7.2	87 570	7.2	99 299	7.1
	Missing	51	<0.1	21	<0.1	30	<0.1
Type of living area^1^	City	1,010,732	38.8	473 572	39.1	537 160	38.5
	Town and suburb	1,094,403	42.0	507 496	41.9	586 907	42.1
	Rural area	500,092	19.2	229 076	18.9	271 016	19.4
Blue-/white-collar work	Blue	1,189,574	45.7	485 826	40.1	703 748	50.4
Occupational prestige (groups)	White	1,415,653	54.3	724 318	59.9	691 335	49.6
	Very high	61,208	5.1	98 801	7.1	160 009	6.1
	High	323,777	26.8	266 180	19.1	589 957	22.6
	Medium	455,239	37.6	460 317	33.0	915 556	35.1
	Low	198,952	16.4	457 888	32.8	656 840	25.2
	Very low	170,968	14.1	111 897	8.0	282 865	10.9

1 Type of living area is based on the degree of urbanisation related to population density [22].

In the cohort, 21% had at least one SA spell in the 3-year follow-up period of 2011–2013 and 5.9% had at least one SA spell >90 days, while 1.6% received part-time or full-time DP. The mean number of SA days/year increased by about 1.5 days per year during the study period (from 4.6 days in 2010 to 9.1 days in 2013).

**Figure 2.**
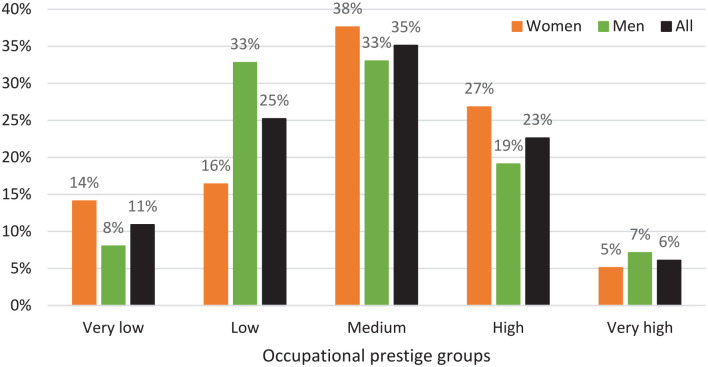
Proportions of women, men and all, respectively, by occupational prestige group.

The distribution by occupational prestige group was 11% in very low, 25% in low, 35% in medium, 23% in high, and 6% in very high (Figure 2). The overall distribution was similar in women and men with the exception that the most common group in women was medium (38%) and medium and low in men (33%). Of the women, 14.1% were in the category ‘very low’ prestige, the corresponding proportion for men was 8.0%.

The mean number of SA days in 2010 varied by occupational prestige group and in all five groups, mean days were somewhat higher among women (Table II).

**Table II.** Mean sickness absence (SA) and disability pension (DP) net days (days with partial SA or DP have been combined to correspond to full days) in 2010 for each occupational prestige group, and *n* (%) with SA spell >14 days 2011–2013, SA >90 days 2011–2013, and DP, respectively, for all as well as for women and men separately.

Occupational prestige (groups)	Mean SA net days 2010	Mean DP net days 2010	*n* (%) with SA >14 days 2011–2013	*n* (%) with SA >90 days 2011–2013	*n* (%) with DP 2011–2013
All					
Very high	2.96	0.88	20,019 (12.5)	5263 (3.3)	1139 (0.7)
High	3.61	1.52	97,522 (16.5)	26,201 (4.4)	7281 (1.2)
Medium	4.58	1.91	194,351 (21.2)	53,720 (5.9)	13,731 (1.5)
Low	5.02	2.20	149,879 (22.8)	41,867 (6.4)	10,802 (1.6)
Very low	6.49	3.43	85,683 (30.3)	26,190 (9.3)	7823 (2.8)
Total	4.58	2.00	547,454 (21.0)	153,241 (5.9)	40,776 (1.6)
Women					
Very high	4.85	1.39	12,210 (19.9)	3216 (5.3)	698 (1.1)
High	4.83	2.18	71,074 (22.0)	19,009 (5.9)	5702 (1.8)
Medium	6.29	2.93	13,1945 (29.0)	37,282 (8.2)	10,440 (2.3)
Low	6.13	4.11	57,347 (28.8)	16,282 (8.2)	5917 (3.0)
Very low	7.41	3.82	60,083 (35.1)	18,487 (10.8)	5240 (3.1)
Total	5.96	2.97	332,659 (27.5)	94,276 (7.8)	27,997 (2.3)
Men					
Very high	1.78	0.57	7809 (7.9)	2047 (2.1)	441 (0.4)
High	2.14	0.72	26,448 (9.9)	7192 (2.7)	1579 (0.6)
Medium	2.89	0.89	62,406 (13.6)	16,438 (3.6)	3291 (0.7)
Low	4.53	1.37	92,532 (20.2)	25,585 (5.6)	4885 (1.1)
Very low	5.09	2.83	25,600 (22.9)	7703 (6.9)	2583 (2.3)
Total	3.38	1.15	214,795 (15.4)	58,965 (4.2)	12,779 (0.9)

**Figure 3.**
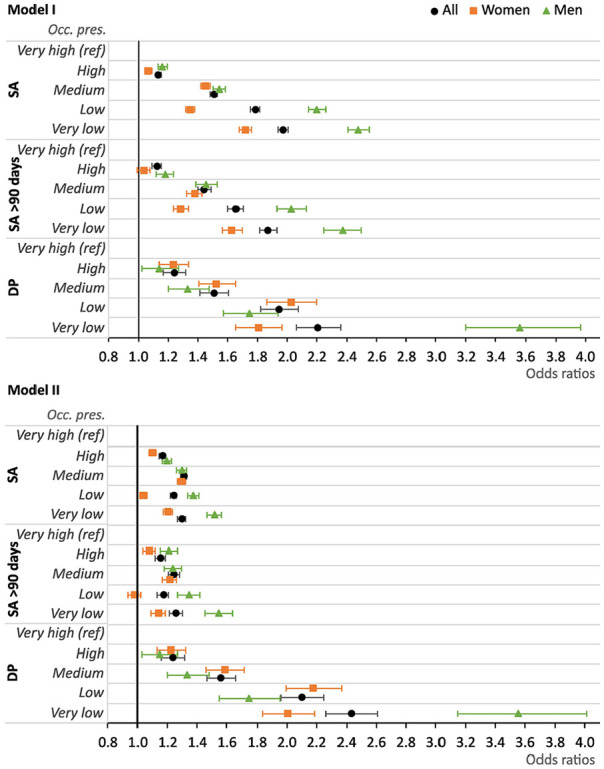
Odds ratios with 95% confidence intervals for occupational prestige (with ‘very high’ as reference) with all three outcomes (SA, SA >90 days, and DP) in the two adjusted models. For all and stratified by sex.

In all logistic regression analyses, the ‘very high’ occupational prestige group was used as reference. The ORs for having at least one SA spell >14 days during the 3-year follow-up were higher for the other four occupational prestige groups compared to the reference group (Table III, Figure 3; see Supplementary Tables 2 and 3 for more information about adjusted ORs) in both the crude and adjusted models. The ORs attenuated somewhat in Model I and more so in Model II, but remained significant.

**Table III.** Odd ratios (ORs) and 95% confidence intervalls (CIs) for all variables in the fully adjusted model (Modell II) for, during follow-up 2011–2013, having at least one SA spell >14 days, >90 days, and for having any disability pension (DP) days. Shown for all, as well as for women and men separately.

		SA spell >14 days			SA spell >90 days			DP		
		All	Women	Men	All	Women	Men	All	Women	Men
		OR (95% CI)	OR (95% CI)	OR (95% CI)	OR (95% CI)	OR (95% CI)	OR (95% CI)	OR (95% CI)	OR (95% CI)	OR (95% CI)
Occupational prestige (groups)	Very high	1	1	1	1	1	1	1	1	1
	High	1.16 (1.14–1.18)	1.10 (1.07–1.12)	1.19 (1.16–1.23)	1.15 (1.12–1.19)	1.07 (1.03–1.11)	1.21 (1.15–1.27)	1.23 (1.16–1.31)	1.22 (1.13–1.32)	1.14 (1.03–1.27)
	Medium	1.31 (1.29–1.33)	1.30 (1.27–1.32)	1.29 (1.26–1.33)	1.24 (1.21–1.28)	1.21 (1.16–1.26)	1.23 (1.18–1.30)	1.56 (1.46–1.66)	1.58 (1.46–1.71)	1.33 (1.20–1.48)
	Low	1.24 (1.22–1.26)	1.04 (1.01–1.06)	1.37 (1.33–1.41)	1.17 (1.13–1.21)	0.98 (0.94–1.02)	1.34 (1.27–1.42)	2.10 (1.96–2.25)	2.17 (2.00–2.37)	1.74 (1.55–1.96)
	Very low	1.29 (1.27–1.32)	1.20 (1.17–1.24)	1.51 (1.47–1.56)	1.25 (1.21–1.30)	1.13 (1.09–1.19)	1.54 (1.46–1.64)	2.43 (2.26–2.61)	2.01 (1.84–2.19)	3.55 (3.15–4.01)
Age	25–34 years	0.89 (0.88–0.90)	1.03 (1.01–1.04)	0.75 (0.74–0.76)	0.76 (0.75–0.77)	0.83 (0.81–0.85)	0.67 (0.65–0.69)	0.23 (0.21–0.24)	0.20 (0.19–0.22)	0.27 (0.24–0.29)
	35–44 years	1	1	1	1	1	1	1	1	1
	45–54 years	1.22 (1.21–1.23)	1.12 (1.11–1.14)	1.34 (1.33–1.36)	1.24 (1.22–1.26)	1.12 (1.10–1.14)	1.43 (1.40–1.46)	2.36 (2.29–2.43)	2.23 (2.15–2.32)	2.59 (2.45–2.73)
	55–59 years	1.52 (1.50–1.54)	1.30 (1.28–1.32)	1.84 (1.81–1.86)	1.62 (1.59–1.65)	1.35 (1.32–1.38)	2.09 (2.04–2.15)	4.25 (4.11–4.40)	3.84 (3.68–4.00)	5.17 (4.87–5.48)
Country of birth	Sweden	1	1	1	1	1	1	1	1	1
	Nordic and EU countries	1.04 (1.03–1.06)	1.03 (1.01–1.04)	1.08 (1.06–1.11)	1.08 (1.05–1.10)	1.06 (1.03–1.09)	1.13 (1.09–1.17)	0.92 (0.88–0.96)	0.92 (0.88–0.97)	0.94 (0.87–1.03)
	Rest of the World	1.16 (1.15–1.18)	1.09 (1.08–1.11)	1.26 (1.24–1.28)	1.24 (1.22–1.26)	1.15 (1.13–1.18)	1.37 (1.33–1.42)	1.01 (0.97–1.05)	0.94 (0.89–0.99)	1.18 (1.10–1.26)
Educational level	Elementary	1	1	1	1	1	1	1	1	1
	High school	1.25 (1.23–1.26)	1.14 (1.12–1.16)	1.43 (1.41–1.46)	1.32 (1.30–1.35)	1.21 (1.18–1.25)	1.56 (1.51–1.60)	1.51 (1.46–1.57)	1.44 (1.38–1.51)	1.71 (1.61–1.82)
	University	1.09 (1.08–1.10)	1.03 (1.02–1.04)	1.29 (1.27–1.31)	1.10 (1.09–1.12)	1.04 (1.02–1.05)	1.33 (1.30–1.37)	1.26 (1.23–1.29)	1.24 (1.20–1.28)	1.39 (1.32–1.47)
Family situation	Married/ cohab. with children	1	1	1	1	1	1	1	1	1
	Married/ cohab. no children	1.11 (1.10–1.12)	1.14 (1.12–1.15)	1.11 (1.09–1.13)	1.09 (1.08–1.11)	1.10 (1.07–1.12)	1.14 (1.11–1.17)	1.65 (1.60–1.70)	1.70 (1.63–1.76)	1.58 (1.50–1.66)
	Single no children	1.16 (1.15–1.17)	1.17 (1.16–1.19)	1.16 (1.15–1.17)	1.26 (1.24–1.28)	1.25 (1.23–1.27)	1.30 (1.27–1.32)	1.81 (1.75–1.86)	1.79 (1.72–1.85)	1.82 (1.74–1.92)
	Single with children	1.34 (1.32–1.36)	1.37 (1.35–1.39)	1.23 (1.19–1.26)	1.47 (1.44–1.50)	1.47 (1.43–1.50)	1.33 (1.27–1.40)	1.24 (1.18–1.31)	1.19 (1.13–1.26)	1.50 (1.33–1.69)
Type of living area	City	1	1	1	1	1	1	1	1	1
	Town and suburb	1.05 (1.05–1.06)	1.05 (1.05–1.06)	1.05 (1.04–1.06)	1.07 (1.06–1.08)	1.08 (1.07–1.10)	1.04 (1.02–1.06)	1.11 (1.08–1.14)	1.10 (1.07–1.13)	1.14 (1.09–1.18)
	Rural area	1.07 (1.06–1.08)	1.06 (1.05–1.07)	1.07 (1.06–1.09)	1.08 (1.07–1.10)	1.09 (1.07–1.11)	1.06 (1.04–1.09)	1.19 (1.16–1.22)	1.15 (1.12–1.19)	1.27 (1.21–1.33)
Blue-/white-collar work	White	1	1	1	1	1	1	1	1	1
	Blue	1.71 (1.70–1.73)	1.62 (1.60–1.64)	1.80 (1.77–1.84)	1.68 (1.65–1.71)	1.65 (1.62–1.68)	1.67 (1.62–1.72)	0.88 (0.86–0.91)	0.87 (0.84–0.90)	1.00 (0.94–1.07)
Sex	Women	2.19 (2.17–2.20)	-	-	1.95 (1.93–1.97)	-	-	2.42 (2.36–2.47)	-	-
	Men	1	-	-	1	-	-	1	-	-

In Model II, the groups with the highest ORs for future SA were those with very low occupational prestige (OR 1.29, 95% CI 1.27–1.32) and those with medium occupational prestige (OR 1.31, 95% CI 1.29–1.33). Among women, the OR was highest for the medium occupational prestige group and second highest for very low occupational prestige (Table III). However, among men, the ORs for future SA followed a gradient: the OR for the group with very low occupational prestige was highest followed by low, medium, and high occupational prestige. Analogous logistic regression analyses as above were carried out for having at least one future SA spell >90 days. The results were comparable to those found for any future SA (see Table III, Figure 3 and Supplementary Tables 4 and 5).

With regard to future DP (Table III, Figure 3, Supplementary Tables 6 and 7), as with the results for SA, the ORs for being granted DP during follow-up were higher for the four occupational prestige groups (‘high’, ‘medium’, ‘low’, and ‘very low’) than for the reference group (‘very high’). This was true for all models (crude, I, and II). Similarly, the results for men followed a gradient from ‘very low’ to ‘high’, with the highest DP OR for those with ‘very low’ occupational prestige and the lowest for those in the group with ‘high’ occupational prestige, however, the pattern was less straightforward for women (Figure 3), as was the case for SA.

Furthermore, the DP OR for those in jobs with ‘very low’ occupational prestige was lower among women than men. For the remaining occupational prestige groups (low, medium and high) this was reversed, that is, the ORs were higher among women than men (Table III). This result differed from the results obtained when analysing the two SA outcomes: in these logistic regressions, lower ORs were generally found among women rather than men.

The OR for future DP was higher for three of the four occupational prestige groups in Model II compared with Model I. This means that adding the variable ‘type of occupation’ (in Model II) did not attenuate the ORs for the occupational prestige groups in the same way as in the case of SA.

With regard to the covariates in Model II for future DP, the ORs were significant for most variables. For birth country (Table III), however, the OR for future DP was neither significantly higher nor lower for people born in ‘rest of the world’ compared to those born in Sweden. Stratifying by sex revealed that women born in countries other than Sweden had a significantly lower OR for future DP than women born in Sweden. For men, another pattern emerged: men in the category ‘rest of the world’ had a higher OR for future DP than men born in Sweden. For the covariate ‘type of occupation’ (blue-/white-collar), the results differed to those for SA. The OR for future DP was significantly lower for blue- than white-collar workers, while the opposite was seen for SA (both in general and in relation to having an SA spell >90 days). When stratified by sex, a significantly lower OR for blue-collar workers for future DP was observed for women but not for men.

## Discussion

In this large population-based prospective cohort study of 2.6 million working people in Sweden, we found an association between occupational prestige scores and future SA/DP during the 3-year follow-up. In general, lower occupational prestige was associated with higher odds of future SA/DP. A clear social gradient was found among men: the lower the occupational prestige, the higher the odds of future SA/DP. Among women, the odds of future SA were highest among those with ‘medium’ occupational prestige and the odds of future DP were highest in the group with ‘low’ occupational prestige.

We found only one previous study, by Nwaru et al., on occupational prestige and SA [7]. They also had a longitudinal design with a 3-year follow-up but did not include information about DP. DP is a more severe outcome than SA, with a definite labour-market exit and, thus, was important to include in the analyses. Furthermore, our cohort was more than twice the size of Nwaru’s, allowing ‘higher resolution’ results regarding occupational prestige group. Nevertheless, our results for SA were in line with their results, with one exception: in Nwaru et al.’s study, a graded association between occupational prestige and SA was found for both women and men [7]. In our study, a distinctly graded association was observed among men, but was not as clear among women. This discrepancy might be attributed to our more detailed categorising of occupational prestige.

The two SA measures (i.e. at least one SA spell >14 days and one >90 days during follow-up) used in both studies yielded very similar results, indicating a similar association between occupational prestige and future shorter and longer SA spells. However, the SA measure ‘>14 days’ contains all spells exceeding 14 days, thus, both shorter and longer spells (i.e. the two measures of SA used in this study) were somewhat related to each other.

The associations between occupational prestige and future SA/DP weakened but were still statistically significant after adjustment for sociodemographic variables. Type of occupation (Model II) attenuated the association for SA but not for DP. This might have been an unnecessary adjustment, reducing both the OR and the precision. Nevertheless, the results remained significant, indicating an independent association with SA and DP. Blue- and white-collar occupations were strongly correlated with occupational prestige; however, blue-/white-collar work and occupational prestige might be two different axes of the association between occupation and SA/DP with different mechanisms underlying the associations. Moreover, such mechanisms might differ between women and men. In future studies, this could be explored further by adding information about the work environment, which might be important in the ascription of prestige.

The social gradient in SA and DP has mainly been explained by detrimental work environments: harmful exposures at work lead to morbidity and reduced work capacity, which in turn lead to SA and even to DP. The corresponding hypothesised process underlying occupational prestige would be an added internalisation of the negative social values. A lower position in the social status gradient can in itself be stressful [23], and belonging to a group ascribed low prestige can through internalisation lead to low self-esteem [3]. Stress and low self-esteem might reduce the use of active coping strategies, for example, changing work or undergoing education to upgrade one’s social position [24,25]. The capability to change life situations is also related to societal possibilities (public benefits for studying) and attitudes (social climate). Future studies of occupational prestige should incorporate analyses of the work environment, which might be a confounder to how occupations are valued and of SA/DP. Research is also needed to explore occupational prestige in relation to different SA/DP diagnoses.

### Strength and limitations

The strengths of this study include the population-based cohort involving all people fulfilling the inclusion criteria, its prospective study design, and the large cohort, allowing subgroup analyses. Our data, from several high-quality registers [26], had low rates of missing values and no dropouts. Moreover, the administrative data was not self-reported with risk of recall bias. Furthermore, we used different measures of SA [21] and included information on DP, which had not been done before in studies of occupational prestige. Only SA spells >14 days were included; this can be seen as both a strength (as temporary illnesses, e.g. colds, did not affect the results) and as a limitation. However, all included people were covered by the same public SA and DP insurance. Another limitation is that SIOPS values were missing for nearly 3% of those in paid work (Figure 1), which is why these individuals were excluded from the analysis.

## Conclusion

Working in lower occupational prestige occupations was generally associated with higher odds of future SA/DP compared to higher prestige occupations: these associations were stronger for men than for women. Furthermore, the social gradient was stronger for men than women. An occupation’s symbolic value and societal esteem might contribute to the social gradient in SA and DP.

## Supplemental Material

sj-docx-1-sjp-10.1177_14034948241272936 – Supplemental material for Occupational prestige and future sickness absence and disability pension in women and men: a Swedish nationwide prospective cohort study Supplemental material, sj-docx-1-sjp-10.1177_14034948241272936 for Occupational prestige and future sickness absence and disability pension in women and men: a Swedish nationwide prospective cohort study by Gunnel Hensing, Mira Müller, Ylva Ulfsdotter Eriksson, Kristina Alexanderson and Kristin Farrants in Scandinavian Journal of Public Health
