# Supplementary material for: Occupational prestige and future sickness absence and disability pension in women and men: a Swedish nationwide prospective cohort study
Source: Scand J Public Health. 2024 Sep 5;53(7):788–96. doi: 10.1177/14034948241272936 (PMC12598064; doi:10.1177/14034948241272936)
Supplement: sj-docx-1-sjp-10.1177_14034948241272936 – Supplemental material for Occupational prestige and future sickness absence and disability pension in women and men: a Swedish nationwide prospective cohort study [file sj-docx-1-sjp-10.1177_14034948241272936.docx]

Suppl table 1. Frequencies and proportions of sociodemographic characteristics of each occupational prestige groups in 2010; for all as well as for women and men separately.

|  | **All** | | | | |  | **Women** | | | | |  | **Men** | | | | |
| --- | --- | --- | --- | --- | --- | --- | --- | --- | --- | --- | --- | --- | --- | --- | --- | --- | --- |
|  | **Very high (6.1%)** | **High (22.6%)** | **Medium (35.1%)** | **Low (25.2%)** | **Very low (10.9%)** |  | **Very high (5.1%)** | **High (26.8%)** | **Medium (37.6%)** | **Low (16.4%)** | **Very low (14.1%)** |  | **Very high (7.1%)** | **High (19.1%)** | **Medium (33.0%)** | **Low (32.8%)** | **Very low (8.0%)** |
|  | n  (%) | n  (%) | n  (%) | n  (%) | n  (%) |  | n  (%) | n  (%) | n  (%) | n  (%) | n  (%) |  | n  (%) | n  (%) | n  (%) | n  (%) | n  (%) |
| **Level of education** |  |  |  |  |  |  |  |  |  |  |  |  |  |  |  |  |  |
| Elementary | 2 022 | 17 194 | 53 017 | 114 737 | 60 902 |  | 244 | 5 749 | 15 693 | 27 536 | 34 930 |  | 1 778 | 11 445 | 37 324 | 87 201 | 25 972 |
|  | (1.3) | (2.9) | (5.8) | (17.5) | (21.5) |  | (0.4) | (1.8) | (3.4) | (13.8) | (20.4) |  | (1.8) | (4.3) | (8.1) | (19.0) | (23.2) |
| High school | 13 468 | 152 399 | 456 120 | 459 677 | 184 625 |  | 2 298 | 71 755 | 223 701 | 137 738 | 114 741 |  | 11 170 | 80 644 | 232 419 | 321 939 | 69 884 |
|  | (8.4) | (25.8) | (49.8) | (70.0) | (65.3) |  | (3.8) | (22.2) | (49.1) | (69.2) | (67.1) |  | (11.3) | (30.3) | (50.5) | (70.3) | (62.5) |
| University/college | 143 754 | 419 351 | 404 931 | 80 193 | 35 654 |  | 58 465 | 245 983 | 215 515 | 33 418 | 20 542 |  | 85 289 | 173 368 | 189 416 | 46 775 | 15 112 |
|  | (89.8) | (71.1) | (44.2) | (12.2) | (12.6) |  | (95.5) | (76.0) | (47.3) | (16.8) | (12.0) |  | (86.3) | (65.1) | (41.1) | (10.2) | (13.5) |
| Missing | 765 | 1 013 | 1 488 | 2 233 | 1 684 |  | 201 | 290 | 330 | 260 | 755 |  | 564 | 723 | 1158 | 1 973 | 929 |
|  | (0.5) | (0.2) | (0.2) | (0.3) | (0.6) |  | (0.3) | (0.1) | (0.1) | (0.1) | (0.4) |  | (0.6) | (0.3) | (0.3) | (0.4) | (0.8) |
| **Family situation** |  |  |  |  |  |  |  |  |  |  |  |  |  |  |  |  |  |
| Married/cohabitant without children | 29 425 | 130 351 | 192 553 | 120 186 | 63 502 |  | 11 668 | 77 840 | 112 763 | 45 622 | 45 450 |  | 17 757 | 52 511 | 79 790 | 74 564 | 18 052 |
|  | (18.4) | (22.1) | (21.0) | (18.3) | (22.4) |  | (19.1) | (24.0) | (24.8) | (22.9) | (26.6) |  | (18.0) | (19.7) | (17.3) | (16.3) | (16.1) |
| Married/cohabitant with children | 78 172 | 265 902 | 381 638 | 246 855 | 92 329 |  | 27 662 | 134 758 | 174 291 | 67 445 | 54 253 |  | 50 510 | 131 144 | 207 347 | 179 410 | 38 076 |
|  | (48.9) | (45.1) | (41.7) | (37.6) | (32.6) |  | (45.2) | (41.6) | (38.3) | (33.9) | (31.7) |  | (51.1) | (49.3) | (45.0) | (39.2) | (34.0) |
| Single without children | 46 680 | 163 290 | 290 046 | 259 823 | 104 989 |  | 18 212 | 87 281 | 128 675 | 67 828 | 52 172 |  | 28 468 | 76 009 | 161 371 | 191 995 | 52 817 |
|  | (29.2) | (27.7) | (31.7) | (39.6) | (37.1) |  | (29.8) | (27.0) | (28.3) | (34.1) | (30.5) |  | (28.8) | (28.6) | (35.1) | (41.9) | (47.2) |
| Single with children | 5 732 | 30 414 | 51 319 | 29 976 | 22 045 |  | 3 666 | 23 898 | 39 510 | 18 057 | 19 093 |  | 2 066 | 6 516 | 11 809 | 11 919 | 2 952 |
|  | (3.6) | (5.2) | (5.6) | (4.6) | (7.8) |  | (6.0) | (7.4) | (8.7) | (9.1) | (11.2) |  | (2.1) | (2.4) | (2.6) | (2.6) | (2.6) |
| **Age** |  |  |  |  |  |  |  |  |  |  |  |  |  |  |  |  |  |
| 25-34 years | 34 598 | 95 195 | 181 690 | 172 390 | 59 204 |  | 15 129 | 50 288 | 76 085 | 46 875 | 29 988 |  | 19 469 | 44 907 | 105 605 | 125 515 | 29 216 |
|  | (21.6) | (16.1) | (19.8) | (26.2) | (20.9) |  | (24.7) | (15.5) | (16.7) | (23.6) | (17.5) |  | (19.7) | (16.9) | (22.9) | (27.4) | (26.1) |
| 35-44 years | 54 168 | 200 172 | 304 987 | 203 204 | 80 324 |  | 20 148 | 107 285 | 146 335 | 62 408 | 48 652 |  | 34 020 | 92 887 | 158 652 | 140 796 | 31 672 |
|  | (33.9) | (33.9) | (33.3) | (30.9) | (28.4) |  | (32.9) | (33.1) | (32.1) | (31.4) | (28.5) |  | (34.4) | (34.9) | (34.5) | (30.7) | (28.3) |
| 45-54 years | 48 631 | 198 851 | 299 411 | 198 010 | 97 727 |  | 17 620 | 111 694 | 162 093 | 62 774 | 62 913 |  | 31 011 | 87 157 | 137 318 | 135 236 | 34 814 |
|  | (30.4) | (33.7) | (32.7) | (30.1) | (34.5) |  | (28.8) | (34.5) | (35.6) | (31.6) | (36.8) |  | (31.4) | (32.7) | (29.8) | (29.5) | (31.1) |
| 55-59 years | 22 612 | 95 739 | 129 468 | 83 236 | 45 610 |  | 8 311 | 54 510 | 70 726 | 26 895 | 29 415 |  | 14 301 | 41 229 | 58 742 | 56 341 | 16 195 |
|  | (14.1) | (16.2) | (14.1) | (12.7) | (16.1) |  | (13.6) | (16.8) | (15.5) | (13.5) | (17.2) |  | (14.5) | (15.5) | (12.8) | (12.3) | (14.5) |
| **Birth country** |  |  |  |  |  |  |  |  |  |  |  |  |  |  |  |  |  |
| Sweden | 135 744 | 542 255 | 826 187 | 574 061 | 216 103 |  | 50 944 | 296 406 | 405 548 | 175 075 | 130 385 |  | 84 800 | 245 849 | 420 639 | 398 986 | 85 718 |
|  | (84.8) | (91.9) | (90.2) | (87.4) | (76.4) |  | (83.2) | (91.5) | (89.1) | (88.0) | (76.3) |  | (85.8) | (92.4) | (91.4) | (87.1) | (76.6) |
| Nordic countries (except Sweden) | 3 758 | 13 605 | 22 088 | 16 721 | 8 339 |  | 1 790 | 8 778 | 13 203 | 6 157 | 5 905 |  | 1 968 | 4 827 | 8 885 | 10 564 | 2 434 |
|  | (2.3) | (2.3) | (2.4) | (2.5) | (2.9) |  | (2.9) | (2.7) | (2.9) | (3.1) | (3.5) |  | (2.0) | (1.8) | (1.9) | (2.3) | (2.2) |
| EU27 (except Denmark, Finland and Sweden) | 8 598 | 11 356 | 17 070 | 13 712 | 8 710 |  | 3 784 | 6 131 | 8 946 | 4 107 | 5 394 |  | 4 814 | 5 225 | 8 124 | 9 605 | 3 316 |
|  | (5.4) | (1.9) | (1.9) | (2.1) | (3.1) |  | (6.2) | (1.9) | (2.0) | (2.1) | (3.2) |  | (4.9) | (2.0) | (1.8) | (2.1) | (3.0) |
| Outside EU27 & the Nordic countries | 11 904 | 22 733 | 50 200 | 52 334 | 49 698 |  | 4 687 | 12 457 | 27 537 | 13 611 | 29 278 |  | 7 217 | 10 276 | 22 663 | 38 723 | 20 420 |
|  | (7.4) | (3.9) | (5.5) | (8.0) | (17.6) |  | (7.7) | (3.8) | (6.0) | (6.8) | (17.1) |  | (7.3) | (3.9) | (4.9) | (8.5) | (18.2) |
| Missing | 5 | 8 | 11 | 12 | 15 |  | 3 | 5 | 5 | 2 | 6 |  | 2 | 3 | 6 | 10 | 9 |
|  | (< 0.1) | (< 0.1) | (< 0.1) | (< 0.1) | (< 0.1) |  | (< 0.1) | (< 0.1) | (< 0.1) | (< 0.1) | (< 0.1) |  | (< 0.1) | (< 0.1) | (< 0.1) | (< 0.1) | (< 0.1) |
| **Type of living area** |  |  |  |  |  |  |  |  |  |  |  |  |  |  |  |  |  |
| Cities | 92 525 | 269 132 | 348 701 | 201 230 | 99 144 |  | 36 541 | 146 649 | 167 076 | 67 547 | 55 759 |  | 55 984 | 122 483 | 181 625 | 133 683 | 43 385 |
|  | (57.8) | (45.6) | (38.1) | (30.6) | (35.0) |  | (59.7) | (45.3) | (36.7) | (34.0) | (32.6) |  | (56.7) | (46.0) | (39.5) | (29.2) | (38.8) |
| Towns and suburbsh | 53 881 | 235 028 | 395 720 | 288 810 | 120 964 |  | 19 791 | 128 909 | 197 913 | 85 983 | 74 900 |  | 34 090 | 106 119 | 197 807 | 202 827 | 46 064 |
|  | (33.7) | (39.8) | (43.2) | (44.0) | (42.8) |  | (32.3) | (39.8) | (43.5) | (43.2) | (43.8) |  | (34.5) | (39.9) | (43.0) | (44.3) | (41.2) |
| Rural areas | 13 603 | 85 797 | 171 135 | 166 800 | 62 757 |  | 4 876 | 48 219 | 90 250 | 45 422 | 40 309 |  | 8 727 | 37 578 | 80 885 | 121 378 | 22 448 |
|  | (8.5) | (14.5) | (18.7) | (25.4) | (22.2) |  | (8.0) | (14.9) | (19.8) | (22.8) | (23.6) |  | (8.8) | (14.1) | (17.6) | (26.5) | (20.1) |
| **Blue/white collar** |  |  |  |  |  |  |  |  |  |  |  |  |  |  |  |  |  |
| Blue | 0 | 472 | 314 602 | 591 722 | 282 778 |  | 0 | 99 | 162 039 | 152 761 | 170 927 |  | 0 | 373 | 152 563 | 438 961 | 111 851 |
|  | 0 | (0.1) | (34.4) | (90.1) | (>99.9) |  | 0 | (<0.1) | (35.6) | (76.8) | (>99.9) |  | 0 | (0.1) | (33.1) | (95.9) | (>99.9) |
| White | 160 009 | 589 485 | 600 954 | 65 118 | 87 |  | 61 208 | 323 678 | 293 200 | 46 191 | 41 |  | 98 801 | 265 807 | 307 754 | 18 927 | 46 |
|  | (100) | (99.9) | (65.6) | (9.9) | (<0.1) |  | (100) | (100) | (64.4) | (23.2) | (<0.1) |  | (100) | (99.9) | (66.9) | (4.1) | (<0.1) |
|  |  |  |  |  |  |  |  |  |  |  |  |  |  |  |  |  |  |
|  |  |  |  |  |  |  |  |  |  |  |  |  |  |  |  |  |  |
| **Total** | 160 009 | 589 957 | 915 556 | 656 840 | 282 865 |  | 61 208 | 323 777 | 455 239 | 198 952 | 170 968 |  | 98 801 | 266 180 | 460 317 | 457 888 | 111 897 |
|  | (100) | (100) | (100) | (100) | (100) |  | (100) | (100) | (100) | (100) | (100) |  | (100) | (100) | (100) | (100) | (100) |

Suppl table 2. Odd ratios (OR) and 95% confidence intervalls (CI) for each of the five occupational prestige groups, using ‘very high’ as reference for having a sickness absence spell >14 days during the follow-up period 2011-2013; crude and adjusted models^1 2^, for all and stratified by sex.

|  | Crude | Model I1 | Model II2 |
| --- | --- | --- | --- |
| **Occupational prestige (groups)** | **OR (95% CI)** | **OR (95% CI)** | **OR (95% CI)** |
|  | **All** |  |  |
| Very high | 1 | 1 | 1 |
| High | 1.39 (1.36–1.41) | 1.14 (1.12-1.15) | 1.16 (1.14-1.18) |
| Medium | 1.88 (1.86–1.91) | 1.51 (1.49-1.53) | 1.31 (1.29-1.33) |
| Low | 2.07 (2.04–2.10) | 1.79 (1.76-1.82) | 1.24 (1.22-1.26) |
| Very low | 3.04 (2.99–3.09) | 1.97 (1.94-2.01) | 1.29 (1.27-1.32) |
|  |  |  |  |
|  | **Women** |  |  |
| Very high | 1 | 1 | 1 |
| High | 1.13 (1.11–1.15) | 1.07 (1.04–1.09) | 1.10 (1.07–1.12) |
| Medium | 1.64 (1.60–1.67) | 1.45 (1.42–1.48) | 1.30 (1.27–1.32) |
| Low | 1.63 (1.59–1.66) | 1.35 (1.32–1.38) | 1.04 (1.01–1.06) |
| Very low | 2.17 (2.13–2.22) | 1.72 (1.68–1.76) | 1.20 (1.17–1.24) |
|  |  |  |  |
|  | **Men** |  |  |
| Very high | 1 | 1 | 1 |
| High | 1.29 (1.25–1.32) | 1.16 (1.13–1.19) | 1.19 (1.16–1.23) |
| Medium | 1.83 (1.78–1.87) | 1.54 (1.51–1.58) | 1.29 (1.26–1.33) |
| Low | 2.95 (2.88–3.02) | 2.20 (2.15–2.26) | 1.37 (1.33–1.41) |
| Very low | 3.46 (3.37–3.55) | 2.48 (2.41–2.55) | 1.51 (1.47–1.56) |

^1^ Adjusted for: age, birth country, educational level, family situation, and type of living area

^2^ Adjusted for the variables in model I and for blue/white collar occupation

Suppl table 3. Odd ratios (OR) and 95% confidence intervalls (CI) for the occupational prestige groups using ‘very high’ as reference for having at least one sickness absence spell > 14 days in 2011-2013 in models with univariate adjustements; for all, as well as for women and men separatly. “Adjusted for age” is, for example, a model in which only the variables occupational prestige and age were entered.

|  | Adjusted for age | Adjusted for sex | Adjusted for country of birth | Adjusted for educational level | Adjusted for family situation | Adjusted for type of living area | Adjusted for Blue/white collar work |
| --- | --- | --- | --- | --- | --- | --- | --- |
| **Occupational prestige (groups)** | **OR (95% CI)** | **OR (95% CI)** | **OR (95% CI)** | **OR (95% CI)** | **OR (95% CI)** | **OR (95% CI)** | **OR (95% CI)** |
|  | **All** |  |  |  |  |  |  |
| Very high | 1 | 1 | 1 | 1 | 1 | 1 | 1 |
| High | 1.35 (1.33–1.37) | 1.22 (1.20-1.24) | 1.39 (1.37–1.42) | 1.33 (1.31–1.35) | 1.36 (1.33–1.38) | 1.36 (1.34–1.39) | 1.38 (1.36–1.41) |
| Medium | 1.88 (1.85–1.91) | 1.74 (1.72-1.77) | 1.89 (1.86–1.92) | 1.71 (1.68–1.73) | 1.84 (1.81–1.87) | 1.84 (1.81–1.87) | 1.53 (1.51–1.56) |
| Low | 2.11 (2.07–2.14) | 2.24 (2.21-2.28) | 2.07 (2.03–2.10) | 1.73 (1.70–1.76) | 2.03 (1.99–2.06) | 2.00 (1.96–2.03) | 1.25 (1.23–1.27) |
| Very low | 3.00 (2.95–3.05) | 2.62 (2.58-2.67) | 3.00 (2.95–3.05) | 2.53 (2.49–2.58) | 2.89 (2.84–2.94) | 2.95 (2.90–3.00) | 1.75 (1.72–1.79) |
|  |  |  |  |  |  |  |  |
|  | **Women** |  |  |  |  |  |  |
| Very high | 1 | - | 1 | 1 | 1 | 1 | 1 |
| High | 1.11 (1.09–1.14) | - | 1.13 (1.11–1.16) | 1.08 (1.06–1.11) | 1.12 (1.09–1.14) | 1.12 (1.09–1.14) | 1.13 (1.10–1.15) |
| Medium | 1.62 (1.58–1.65) | - | 1.64 (1.61–1.67) | 1.48 (1.45–1.51) | 1.61 (1.57–1.64) | 1.61 (1.57–1.64) | 1.34 (1.31–1.37) |
| Low | 1.62 (1.59–1.66) | - | 1.63 (1.59–1.66) | 1.36 (1.33–1.39) | 1.58 (1.54–1.61) | 1.59 (1.56–1.63) | 1.07 (1.05–1.10) |
| Very low | 2.13 (2.08–2.18) | - | 2.15 (2.11–2.20) | 1.79 (1.75–1.83) | 2.09 (2.05–2.14) | 2.12 (2.08–2.17) | 1.29 (1.25–1.32) |
|  |  |  |  |  |  |  |  |
|  | **Men** |  |  |  |  |  |  |
| Very high | 1 | - | 1 | 1 | 1 | 1 | 1 |
| High | 1.27 (1.23–1.30) | - | 1.29 (1.26–1.33) | 1.16 (1.13–1.20) | 1.28 (1.24–1.31) | 1.26 (1.23–1.30) | 1.28 (1.25–1.32) |
| Medium | 1.89 (1.84–1.93) | - | 1.84 (1.79–1.88) | 1.49 (1.45–1.53) | 1.82 (1.77–1.86) | 1.78 (1.74–1.82) | 1.37 (1.34–1.41) |
| Low | 3.11 (3.03–3.19) | - | 2.95 (2.87–3.02) | 2.08 (2.03–2.14) | 2.92 (2.85–2.99) | 2.82 (2.75–2.89) | 1.46 (1.42–1.50) |
| Very low | 3.56 (3.47–3.66) | - | 3.40 (3.31–3.49) | 2.44 (2.37–2.51) | 3.39 (3.30–3.49) | 3.36 (3.27–3.45) | 1.67 (1.62–1.72) |

Suppl table 4. Odd ratios (OR) and 95% confidence intervalls (CI) for each of the five occupational prestige groups, using ‘very high’ as reference for having a sickness absence spell >90 days during the follow-up period 2011-2013; crude and adjusted models^1 2^, for all and stratified by sex.

|  | Crude | Model I1 | Model II2 |
| --- | --- | --- | --- |
| **Occupational prestige (groups)** | **OR (95% CI)** | **OR (95% CI)** | **OR (95% CI)** |
|  | **All** |  |  |
| Very high | 1 | 1 | 1 |
| High | 1.37 (1.33–1.41) | 1.12 (1.09-1.16) | 1.15 (1.12-1.19) |
| Medium | 1.83 (1.78–1.89) | 1.44 (1.40-1.49) | 1.24 (1.21-1.28) |
| Low | 2.00 (1.94–2.06) | 1.65 (1.60-1.71) | 1.17 (1.13-1.21) |
| Very low | 3.00 (2.91–3.09) | 1.87 (1.81-1.93) | 1.25 (1.21-1.30) |
|  |  |  |  |
|  | **Women** |  |  |
| Very high | 1 | 1 | 1 |
| High | 1.13 (1.08–1.17) | 1.04 (1.00–1.08) | 1.07 (1.03–1.11) |
| Medium | 1.61 (1.55–1.67) | 1.38 (1.33–1.43) | 1.21 (1.16–1.26) |
| Low | 1.61 (1.55–1.67) | 1.29 (1.24–1.34) | 0.98 (0.94–1.02) |
| Very low | 2.19 (2.10–2.27) | 1.63 (1.57–1.70) | 1.13 (1.09–1.19) |
|  |  |  |  |
|  | **Men** |  |  |
| Very high | 1 | 1 | 1 |
| High | 1.31 (1.25–1.38) | 1.18 (1.12–1.24) | 1.21 (1.15–1.27) |
| Medium | 1.75 (1.67–1.83) | 1.46 (1.39–1.53) | 1.23 (1.18–1.30) |
| Low | 2.80 (2.67–2.93) | 2.03 (1.93–2.13) | 1.34 (1.27–1.42) |
| Very low | 3.49 (3.33–3.67) | 2.37 (2.25–2.50) | 1.54 (1.46–1.64) |

^1^ Adjusted for age, country of birth, educational level, family situation, and type of living area

^2^ Adjusted for all variables in model I and for white/blue collar occupation

Suppl table 5. Odd ratios (OR) and 95% confidence intervalls (CI) for the occupational prestige groups using ‘very high’ as reference for having a sickness absence spell >90 days during the follow-up period 2011-2013 in models with univariate adjustements; for all, as well as for women and men separatly. “Adjusted for age” is, e.g., a model in which only the variables occupational prestige and age were entered.

|  | Adjusted for age | Adjusted for sex | Adjusted for country of birth | Adjusted for educational level | Adjusted for family situation | Adjusted for type of living area | Adjusted for Blue/white collar work |
| --- | --- | --- | --- | --- | --- | --- | --- |
| **Occupational prestige (groups)** | **OR (95% CI)** | **OR (95% CI)** | **OR (95% CI)** | **OR (95% CI)** | **OR (95% CI)** | **OR (95% CI)** | **OR (95% CI)** |
|  |  |  |  |  |  |  |  |
|  | **All** |  |  |  |  |  |  |
| Very high | 1 | 1 | 1 | 1 | 1 | 1 | 1 |
| High | 1.32 (1.28–1.36) | 1.22 (1.19-1.26) | 1.38 (1.34–1.42) | 1.30 (1.26–1.34) | 1.34 (1.30–1.38) | 1.35 (1.31–1.39) | 1.37 (1.33–1.41) |
| Medium | 1.82 (1.77–1.87) | 1.70 (1.65-1.75) | 1.84 (1.79–1.90) | 1.62 (1.58–1.67) | 1.78 (1.73–1.83) | 1.79 (1.73–1.84) | 1.45 (1.41–1.50) |
| Low | 2.05 (1.99–2.11) | 2.13 (2.07-2.19) | 2.00 (1.94–2.06) | 1.61 (1.56–1.66) | 1.94 (1.89–2.00) | 1.93 (1.87–1.99) | 1.18 (1.14–1.22) |
| Very low | 2.94 (2.85–3.03) | 2.61 (2.53-2.69) | 2.95 (2.86–3.04) | 2.39 (2.31–2.47) | 2.82 (2.73–2.90) | 2.91 (2.82–3.00) | 1.69 (1.63–1.75) |
|  |  |  |  |  |  |  |  |
|  | **Women** |  |  |  |  |  |  |
| Very high | 1 | - | 1 | 1 | 1 | 1 | 1 |
| High | 1.09 (1.05–1.13) | - | 1.13(1.09–1.18) | 1.07 (1.03–1.11) | 1.11 (1.07–1.15) | 1.11 (1.07–1.15) | 1.12 (1.08–1.17) |
| Medium | 1.56 (1.51–1.62) | - | 1.61(1.56–1.67) | 1.42 (1.37–1.48) | 1.57 (1.52–1.63) | 1.57 (1.51–1.63) | 1.28 (1.23–1.33) |
| Low | 1.60 (1.54–1.66) | - | 1.61(1.55–1.67) | 1.29 (1.24–1.35) | 1.55 (1.49–1.62) | 1.56 (1.50–1.62) | 1.02 (0.98–1.07) |
| Very low | 2.11 (2.03–2.20) | - | 2.16(2.07–2.24) | 1.72 (1.65–1.79) | 2.09 (2.01–2.18) | 2.12 (2.04–2.20) | 1.25 (1.20–1.30) |
|  |  |  |  |  |  |  |  |
|  | **Men** |  |  |  |  |  |  |
| Very high | 1 | - | 1 | 1 | 1 | 1 | 1 |
| High | 1.29 (1.22–1.35) | - | 1.33(1.26–1.39) | 1.18 (1.12–1.24) | 1.30 (1.24–1.37) | 1.29 (1.23–1.36) | 1.31 (1.25–1.38) |
| Medium | 1.81 (1.73–1.90) | - | 1.76(1.68–1.85) | 1.40 (1.33–1.47) | 1.73 (1.65–1.81) | 1.71 (1.63–1.79) | 1.33 (1.26–1.39) |
| Low | 2.95 (2.82–3.09) | - | 2.79(2.67–2.92) | 1.91 (1.82–2.01) | 2.73 (2.60–2.85) | 2.69 (2.56–2.81) | 1.44 (1.37–1.52) |

Suppl table 6. Odd ratios (OR) and 95% confidence intervalls (CI) for each of the five occupational prestige groups, using ‘very high’ as reference for disability pension during the follow-up period 2011-2013; crude and adjusted models^1 2^, for all and stratified by sex.

|  | **Crude** |  | **Model I^1^** |  | **Model II^2^** |
| --- | --- | --- | --- | --- | --- |
| **Occupational prestige (groups)** | **OR (95% CI)** |  | **OR (95% CI)** |  | **OR (95% CI)** |
|  | **All** |  |  |  |  |
| Very high | 1 |  | 1 |  | 1 |
| High | 1.74 (1.64–1.86) |  | 1.24 (1.16-1.32) |  | 1.23 (1.16-1.31) |
| Medium | 2.12 (2.00–2.26) |  | 1.51 (1.42-1.61) |  | 1.56 (1.46-1.66) |
| Low | 2.33 (2.19–2.48) |  | 1.94 (1.82-2.08) |  | 2.10 (1.96-2.25) |
| Very low | 3.97 (3.73–4.22) |  | 2.20 (2.06-2.36) |  | 2.43 (2.26-2.61) |
|  |  |  |  |  |  |
|  | **Women** |  |  |  |  |
| Very high | 1 |  | 1 |  | 1 |
| High | 1.55 (1.44–1.68) |  | 1.23 (1.14–1.34) |  | 1.22 (1.13–1.32) |
| Medium | 2.04 (1.88–2.20) |  | 1.53 (1.41–1.65) |  | 1.58 (1.46–1.71) |
| Low | 2.66 (2.46–2.88) |  | 2.03 (1.86–2.20) |  | 2.17 (2.00–2.37) |
| Very low | 2.74 (2.53–2.97) |  | 1.80 (1.66–1.96) |  | 2.01 (1.84–2.19) |
|  |  |  |  |  |  |
|  | **Men** |  |  |  |  |
| Very high | 1 |  | 1 |  | 1 |
| High | 1.33 (1.20–1.48) |  | 1.14 (1.03–1.27) |  | 1.14 (1.03–1.27) |
| Medium | 1.61 (1.45–1.77) |  | 1.33 (1.20–1.48) |  | 1.33 (1.20–1.48) |
| Low | 2.41 (2.18–2.65) |  | 1.74 (1.57–1.94) |  | 1.74 (1.55-1.96) |
| Very low | 5.27 (4.76–5.83) |  | 3.56 (3.19–3.97) |  | 3.55 (3.15-4.01) |

^1^ Adjusted for age, country of birth, educational level, family situation, and type of living area

^2^ Adjusted for all variables in model II and for white/blue collar occupation

Suppl table 7. Odd ratios (OR) and 95% confidence intervalls (CI) for the occupational prestige groups using ‘very high’ as reference for disability pension in 2011-2013 in models with univariate adjustements; for all, as well as for women and men separatly. “Adjusted for age” is, for example, a model in which only the variables occupational prestige and age were entered.

|  | **Adjusted for age** | **Adjusted for sex** | **Adjusted for country of birth** | **Adjusted for educational level** | **Adjusted for family situation** | **Adjusted for region of living** | **Adjusted for Blue/white collar work** |
| --- | --- | --- | --- | --- | --- | --- | --- |
| **Occupational prestige (groups)** | **OR (95% CI)** | **OR (95% CI)** | **OR (95% CI)** | **OR (95% CI)** | **OR (95% CI)** | **OR (95% CI)** | **OR (95% CI)** |
|  | **All** |  |  |  |  |  |  |
| Very high | 1 | 1 | 1 | 1 | 1 | 1 | 1 |
| High | 1.59 (1.49–1.69) | 1.50 (1.41-1.59) | 1.72 (1.61–1.83) | 1.62 (1.52–1.72) | 1.65 (1.55–1.76) | 1.68 (1.58–1.79) | 1.74 (1.64–1.86) |
| Medium | 2.08 (1.96–2.21) | 1.91 (1.80-2.03) | 2.10 (1.98–2.23) | 1.78 (1.67–1.89) | 2.00 (1.88–2.12) | 2.00 (1.89–2.13) | 2.16 (2.03–2.30) |
| Low | 2.48 (2.34–2.64) | 2.54 (2.39-2.70) | 2.32 (2.18–2.47) | 1.69 (1.58–1.80) | 2.19 (2.06–2.33) | 2.15 (2.02–2.28) | 2.44 (2.28–2.61) |
| Very low | 3.70 (3.48–3.94) | 3.26 (3.06-3.47) | 4.04 (3.79–4.30) | 2.82 (2.64–3.01) | 3.53 (3.31–3.76) | 3.70 (3.48–3.94) | 4.18 (3.90–4.48) |
|  |  |  |  |  |  |  |  |
|  | **Women** |  |  |  |  |  |  |
| Very high | 1 | - | 1 | 1 | 1 | 1 | 1 |
| High | 1.34 (1.23–1.45) | - | 1.53 (1.41–1.65) | 1.41 (1.30–1.53) | 1.48 (1.37–1.60) | 1.50 (1.38–1.62) | 1.55 (1.44–1.68) |
| Medium | 1.79 (1.66–1.94) | - | 2.01 (1.86–2.17) | 1.63 (1.51–1.76) | 1.90 (1.76–2.05) | 1.92 (1.77–2.07) | 2.04 (1.89–2.20) |
| Low | 2.62 (2.42–2.84) | - | 2.63 (2.43–2.85) | 1.81 (1.66–1.96) | 2.45 (2.26–2.65) | 2.48 (2.29–2.68) | 2.67 (2.46–2.90) |
| Very low | 2.33 (2.15–2.53) | - | 2.80 (2.58–3.03) | 1.78 (1.64–1.93) | 2.46 (2.27–2.66) | 2.55 (2.35–2.76) | 2.76 (2.53–3.01) |
|  |  |  |  |  |  |  |  |
|  | **Men** |  |  |  |  |  |  |
| Very high | 1 | - | 1 | 1 | 1 | 1 | 1 |
| High | 1.27 (1.15–1.42) | - | 1.31 (1.18–1.46) | 1.17 (1.05–1.30) | 1.30 (1.17–1.44) | 1.28 (1.15–1.42) | 1.33 (1.20–1.48) |
| Medium | 1.72 (1.56–1.90) | - | 1.59 (1.44–1.75) | 1.23 (1.11–1.36) | 1.56 (1.41–1.73) | 1.51 (1.37–1.67) | 1.47 (1.33–1.63) |
| Low | 2.67 (2.42–2.94) | - | 2.39 (2.16–2.63) | 1.51 (1.36–1.67) | 2.29 (2.07–2.52) | 2.17 (1.96–2.39) | 1.89 (1.69–2.12) |
| Very low | 5.48 (4.95–6.07) | - | 5.33 (4.81–5.90) | 3.24 (2.91–3.61) | 4.89 (4.42–5.42) | 4.92 (4.45–5.45) | 4.11 (3.64–4.63) |
